# A descriptive cross sectional study comparing barriers and determinants of physical activity of Sri Lankan middle aged and older adults

**DOI:** 10.1371/journal.pone.0232956

**Published:** 2020-05-12

**Authors:** A. L. Karunanayake, C. D. Senaratne, A. Stathi

**Affiliations:** 1 Department of Anatomy, Faculty of Medicine, University of Kelaniya, Kelaniya, Sri Lanka; 2 Department of English Language Teaching, University of Kelaniya, Kelaniya, Sri Lanka; 3 Department of Health, University of Bath, Bath, England, United Kingdom; University of Colombo Faculty of Medicine, SRI LANKA

## Abstract

**Background:**

Benefits of physical activities are numerous. Barriers for physical exercise may differ among middle aged and older adults. Therefore, identifying and comparing the barriers for participating in regular physical exercises among middle aged and older adults will be useful in designing age specific physical exercise programmes.

**Methods:**

This descriptive cross sectional study was carried out among 206 Sri Lankan adults in the age range of 40–84 years in the Colombo North region of Sri Lanka using culturally validated questionnaires to determine and compare the barriers and factors associated with regular physical activity participation. Majority were males (56%) and 54% were < 60 years. People in the age range of 40–59 years were considered as middle age and ≥ 60 years as older adults. Bivariate analysis and multivariate analysis was carried out to determine the significant factors that are associated with regular physical activity participation.

**Results:**

Lack of free time (52%), feeling too lazy (26%) and bad weather (29%) were the main barriers for the participants. In < 60 years, high level of income (p = 0.008) and in ≥ 60 years, being a male (p = 0.016), having a high level of education (P = 0.002) and a high BMI (p = 0.002) had a significant negative association with the level of physical activities.

**Conclusions:**

Contrary to findings from surveys in several developed countries, this study showed that having a high level of education and being a male were strongly related with lack of physical activity participation.

## Introduction

Many studies have demonstrated that physical activity has a positive association with quality of life, physical capacity and cardio respiratory fitness. [1] Regular participation in physical exercise has helped minimally disabled patients with multiple sclerosis to remain active and maintain their independence. [2] A study conducted by Pereira et al (2019) demonstrates that participating in regular physical exercise is beneficial to people with bipolar disorders. [3] According to Romero et al (2017) lifestyle intervention is effective in treating non-alcoholic fatty liver disease (NAFLD) patients and weight reductions of more than 10% can induce a significant improvement in non-alcoholic steato hepatitis and fibrosis. [4] Physical exercise either in the form of aerobic or resistance training is known to significantly improve nonalcoholic fatty liver disease (NAFLD). [5]

Even though the importance of physical activity is well known, an alarming percentage (30%) of people throughout the world are physically inactive. [6] Studies have demonstrated that there are many types of environmental, social and personal barriers for the regular participation in physical activity.

### Environmental barriers

Women living in socioeconomically disadvantaged neighborhoods in Australia are at an increased risk of physical inactivity due to less access to facilities and higher levels of crime compared to people living in more advantaged neighborhoods. [7] Poor climatic conditions, was a major barrier to physical activity for men and women both in the 40–60 year age group. [8] Tucker and Gilliand (2007) mentions that level of physical activity varies during different seasons and by providing indoor facilities for physical exercise can promote physical activity during cold and wet seasons. [9] Baert et al (2011) mention that attending to fears and preferences related to physical exercise physical activity can be promoted among people in the age range of 80 years and above. [10] Matters related to safety and bad weather does not demonstrate a significant association with the physical activity levels. [11]

### Social barriers

Research conducted in the United Kingdom, the United States, Canada, New Zealand and Australia has revealed that physical activity levels of South Asians are lower than the Europeans. Major barriers for physical activity mentioned in those studies were cultural differences with the dominant society and difficulties in communicating in English. [12] Fear of breaking cultural norms, lack of culturally sensitive facilities, lack of familiarization with their local neighborhood and difficulties in speaking English were the main barriers for regular physical activity participation for female respondents in the age range of 40–60 years. [8] By improving the social support available for citizens in the age range of 80 years and above can improve their level of physical activities. [10] According to a systematic review done by Humpel et al (2002), aesthetic attributes had a significant association with levels of physical activity. [11]

### Personal barriers

A study conducted in the United States finds that people who prefer sedentary behavior are prone to be physically inactive. [13] Lack of time and money have also been cited as personal barriers. [12] A study done in the United Kingdom mentions that Indians and Pakistanis in the age range of 40 to 60 years in Britain, due to working very long hours in shops and restaurants were the key barriers for them not to take part in activities like walking and swimming on a regular basis. [8] Humple and Lesli (2002) after reviewing nineteen articles that studied the relationship between physical activity and behavior mentions that providing facilities and opportunities can significantly improve the level of physical activities. [11] Baert et al (2011) after studying forty-four articles on barriers and motivators for physical activity mentions that to promote regular physical activity among people in the age range of 80 years and above is important to pay special attention to their health benefits. [10]

The above mentioned studies done in other countries have included people in the age range of 40–60 years and above 80 years. None of the studies have compared the levels of physical activities and barriers for physical activities among people less than 60 year old and equal to or more than 60 years.

A descriptive study conducted on thirty Sri Lankan young adults between the ages of 18–30 years indicates that lack of time due to educational engagements after working hours for under graduate and post graduate education, watching television, reading books and lack of interest in exercises are barriers for regular physical activity participation. [14] This study does not include the 40–60 year age group and above 60 year age group. There were no other studies conducted in Sri Lanka with regard to barriers for regular physical activity participation. Twenty percent of Sri Lankan adults are affected with either diabetes or pre diabetes and 30% affected with diabetes are undiagnosed. [15] Type 2 diabetes mellitus has reached epidemic proportions in Asian countries. A study done on South Asian women aged 30 to 45 years in Western province of Sri Lanka have shown that physical in activity and sedentary behavior contributed to dysglycaemia after adjustment for family history, diet, systolic blood pressure and Body Mass Index. The current global guidelines on physical activity recommend 150 minutes of moderate intensity physical activity per week to achieve substantial health benefits. [16] Since the prevalence of non communicable diseases are high in Sri Lanka and not many studies have been done to determine the levels of physical activities and barriers for participation in regular physical activities, the present study was carried out to compare the levels of physical activity participation, personal, environmental and social barriers for regular physical activity participation and the other factors associated with regular physical activity participation among people less than 60 years and equal to or above 60 years. Our study findings will be useful in designing age specific and culturally suitable physical exercise programmes for middle age and older adults.

## Materials & methods

### Ethics statement

The protocol for the research project was specifically approved by University of Bath Department for Health Research Ethics Approval Committee for Health United Kingdom. The study was conducted performed in accordance with the ethical standards laid down in an appropriate version of the Declaration of Helsinki (as revised in Brazil 2013). In addition to obtaining ethical approval, prior to conducting this study the approvals were also obtained by the management committees of the two Sri Lankan Family Medicine Clinics (Colombo North Family Physicians & General Practitioners Centre Sri Lanka and Colombo North Family Medicine Centre Sri Lanka). All patients gave their informed written consent prior to their inclusion in the study.

A descriptive cross sectional study was carried out in two of the large Family Medicine Clinics in the Colombo North region of the Western province which is 24 km away from Colombo, the capital of Sri Lanka.

### Sample size calculation

From the two clinic registers, patients who are above or equal to the age of 40 years was selected. Accordingly, there were 544 patients. Out of them, the patients who fell into the exclusion criteria (severe ischemic heart disease, heart failure, cerebro vascular accidents and severe hypertension were excluded. After excluding the people who had the exclusion criteria (8%), the remaining people were considered as the target population of the two clinics (500). With the help of the Morgan’s Table (Krejcie and Morgan, 1970) [17] the sample size for the target population was calculated. According to the table when a confidence level of 95% and a margin of error of 5%, is used the sample size for 500 target population is 217. The target population and the sample size were used to calculate the number of patients that was selected from each clinic. Thereafter, using random numbers from random number tables, patients were selected from each clinic.

#### Measures

Culturally validated structured interviewer administered questionnaires were used to collect data. The physical activity level of the subjects was evaluated using the International Physical Activity Questionnaire (IPAQ) culturally validated for Sri Lanka used in the study by Perera et al (2017). [18] The barriers for physical activity were assessed using a questionnaire culturally validated for Sri Lanka used by (Goonawardene, 1996). [14]

These questionnaires consisted of questions related to demographic data, medical and surgical history, preferences for types of physical activity and perceived personal, environmental and social barriers for participation in physical activity. X number of questions assessed personal, environmental and social barriers on a five-point Likert scale. [14] The amount of physical activity done was assessed according to the number of days per week and the minutes spent per day on vigorous, moderate and light intensity activities. The physical activities were categorized into vigorous, moderate and light, based on guidelines of IPAQ. [19] The total duration of activities (high and moderate intensity) done per week with regard to gardening, household activities, recreational activities and sport were calculated by adding the total amount of moderate and vigorous intensity physical activities done during the week.

To determine the association between level of physical activity participation and age, gender, level of education, level of income, total barriers and BMI, bivariate analyses and multivariate analyses (linear regression) were conducted. The p values and confidence intervals were used to assess the level of significance.

The age was divided into two categories. People in the age range of 40–59 years were considered as middle age and ≥ 60 years as older adults. Although the cutoff age for the elderly in many developed countries is considered as 65 years or above, to suite many countries in the world, the United Nations have agreed on the cut-off of age to be 60 years or more for the older population (Satharasinghe, 2016) [20]. In Sri Lanka too, people who are 60 years or more are considered as the elderly or as the older population (Satharasinghe, 2016) [20]. Accordingly, this study defines people who are 60 years or more as the elderly or the older population.

The level of education, level of income and BMI were divided into two groups according to the study done by Karunanayake et al (2006). [21] The level of education two groups were people who had completed tertiary education and people who had less than tertiary education. The level of income two groups were high (≥272$) and low (<272$). BMI two groups were high (≥25) and normal (< 25).

Barriers were divided into two groups that were people having (≤ 1) barrier and who have more than one barrier.

Age, gender, BMI, level of income, level of education and the number of barriers were included in the bivariate and multivariate analyses (linear regression).

The data were analyzed using SPSS version 21. Categorical data were described using percentages and continuous data were described using means and standard deviations.

## Results

Out of 217 adults only 206 took part in the study. Out of these 206 adults 111 were in < 60 year age group and 95 were in ≥ 60 year age group. The age range of participants was 40–84 years. In <60 year age group, 59% were males and the mean age (SD) was 48.9 (5.7) years. In ≥ 60 year group, 54% were males and the mean age (SD) was 69.5 (7.4) years.

### Level of physical activities

In <60 year group, 34% of males were involved in vigorous level of physical activities and the mean level of vigorous activities/week was 99.8 minutes. In this group, none of the females were involved in vigorous activities. With regard to moderate level of physical activities, 96% of females were involved in moderate level of physical activities and their mean level of moderate physical activities/week was 470.7 minutes. With regard to males, only 62% were involved in moderate level of physical activities and their mean level of moderate physical activities were 264.4 minutes/week. In this group, 37% of the participants did not obtain 150 minutes/week of moderate intensity physical activities (Table 1).

**Table 1:** Levels of physical activities, income, education and BMI of study participants.

Levels of Physical Activities	Females ≥60 yrs. (n = 44)		Females <60 yrs. (n = 46)		Males ≥60 yrs. (n = 51)		Males <60 yrs. (n = 65)		Total % (n = 206)	
	Number	%	Number	%	Number	%	Number	%	Number	%
Moderate Intensity Activities	35	80	44	96	37	73	40	62	156	76
Vigorous Intensity Activities	2	5	0	0	4	8	22	34	28	14
**Level of Income**										
Income ≥ 272$	21	47	19	41	26	51	32	49	98	48
Income < 272$	23	53	27	59	25	49	33	51	108	52
**Level of Education**										
< Tertiary Education	20	45	29	63	35	69	34	52	118	57
≥ Tertiary Education	24	55	17	37	16	31	31	48	88	43
**BMI**										
BMI ≥ 25	20	45	30	65	22	43	46	71	118	57
BMI < 25	24	55	16	35	29	57	19	29	88	43

In ≥ 60 year group, 8% of males were involved in vigorous level of physical activities and the mean level of vigorous activities/week was 26.7 minutes. In this group only 5% of females were involved in vigorous activities and the mean level of vigorous activities/week was 8 minutes. With regard to moderate level of physical activities, 80% of females were involved in moderate level of physical activities and their mean level of moderate physical activities were 471 minutes/week. With regard to males, only 73% were involved in moderate level of physical activities and their mean level of moderate physical activities were 249.2 minutes/week. In this group 44% of people did not get 150 minutes/week of moderate intensity physical activities (Table 1).

### Level of income

In <60 year group, 41% of females and 49% of males had a monthly income of more than 272$. In ≥ 60 year group 47% of females and 51% of males had a monthly income of more than 272$.

### Level of education (Table 1)

In <60 year group, 37% of females and 48% of males, had reached the tertiary education level and in ≥ 60 year group, 55% of females and 31% of males, had reached the tertiary education level (Table 1).

### BMI

In <60 year group, the mean BMI (SD) was 27.0 (4.0) Kg and 65% of females and 71% of males had a BMI ≥ 25. In ≥ 60 year group, the mean BMI (SD) was 24.7 (3.8) Kg and 45% of females and 43% of males had a BMI ≥ 25 (Table 1).

### Barriers for participation in regular physical activities

In <60 year age group, 96% of females and 49% of males cited lack of free time as the main personal barrier to take part in leisure time physical activities and 17% of females and 34% of males cited bad weather as the main environmental barrier to take part in leisure time physical activities. Social barriers were not cited by any of the participants.

In ≥ 60 year age group, 55% of females cited lack of free time as the main personal barrier and 37% of males cited feeling too lazy as the main personal barrier to take part in leisure time physical activities. Bad weather was mentioned as the main environmental barrier to take part in leisure time physical activities by 36% of females and 27% of males. Seven percent of the females and 2% of the males mentioned only one type of social barrier, which is that they did not like being seen by others, while being involved in regular leisure time physical activities (Table 2).

**Table 2:** Barriers for regular physical activity participation among males and females age< 60 yrs. and age ≥60 yrs.

Type of Barrier	Females ≥60 yrs. (n = 44)		Females <60 yrs. (n = 46)		Males ≥60 yrs. (n = 51)		Males <60 yrs. (n = 65)		Total (n = 206)	%
Personal	Number	%	Number	%	Number	%	Number	%	Number	%
Lack of Discipline	0	0	0	0	2	4	4	6	6	3
Lack of free time	24	55	44	96	7	14	32	49	107	52
Lack of money	0	0	0	0	0	0	4	6	4	2
Major life event	0	0	0	0	0	0	2	3	2	1
Feeling too tired	4	9	7	15	2	4	4	6	17	8
Feeling too lazy	15	34	11	24	19	37	8	12	53	26
Lack of belief	2	5	0	0	0	0	0	0	2	1
Lack of enjoyment	0	0	0	0	2	4	0	0	2	1
Lack of support	0	0	2	4	4	8	0	0	6	3
Lack of friends	10	23	0	0	3	6	0	0	13	6
Fear of Leg pains	3	7	2	4	4	8	0	0	9	4
Feels Enough exercise, So no need for more	5	11	2	4	0	0	2	3	9	4
Fear of falling	4	9	0	0	9	18	0	0	13	6
**Environmental barriers**										
Bad weather	16	36	8	17	14	27	22	34	60	29
Hazards from thieves, unleashed dogs and snakes	0	0	0	0	0	0	6	9	6	3
Lack of facilities	3	7	4	9	0	0	4	6	11	5
**Social barriers**										
Did not like seeing by others	3	7	0	0	1	2	0	0	4	2

According to the results of bivariate analysis in <60 year age group the level of income (P = <0.001) and level of education (P = <0.001) had a significant negative association with the level of physical activities and in ≥ 60 year age group, the level of income (P = < 0.001), level of education (P = 0.001) and BMI (P = 0.004) had a significant negative association with the level of physical activities (Table 3).

**Table 3:** Results of bivariate analysis of factors associated with physical exercise. Males and females age< 60 yrs. and age ≥60 yrs.

**Males and Females < 60 yrs. (n = 111)**		
**Variable**	**Pearson Correlation**	**Significance**
Age	0.187	0.050
Gender	-0.116	0.226
BMI	-0.098	0.306
Level of Income	-0.422	0.000
Level of Education	-0.395	0.000
Total number of barriers	0.069	0.470
**Males and Females ≥ 60 yrs. (n = 95)**		
Age	-0.157	0.129
Gender	-0.193	0.061
BMI	-0.291	0.004
Level of Income	-0.416	0.000
Level of Education	-0.386	0.000
Total number of barriers	0.070	0.503

According to the results of multivariate analysis (linear regression), in <60 year age group, only, high level of income (P = 0.008) had a significant negative association with the level of physical activities and in ≥60 year age group, having a high level of education (P = 0.002), high BMI (P = 0.002) and being a male (P = 0.016) had a significant negative association with the level of physical activities (Table 4).

**Table 4:** Results of multivariate analysis of factors associated with physical exercise. Males and females age< 60 yrs. and age ≥ 60 yrs.

**Males and Females < 60 yrs. (n = 111)**				
**Variable**	**B Coefficient**	**Significance**	**95% Confidence Interval**	
			**Lower**	**Upper**
Age	0.059	0.533	-10.5	20.3
Sex	-0.035	0.701	-198.6	134.1
BMI	-0.094	0.285	-30.1	8.9
Level of Income	-0.329	0.008	-519.4	-78.0
Level of Education	-0.141	0.247	-348.9	90.7
Total number of barriers	0.087	0.352	-56.7	157.8
**Males and Females ≥ 60 yrs. (n = 95)**				
Age	-0.151	0.112	-26.1	2.8
Sex	-0.227	0.016	-430.9	-46.0
BMI	-0.321	0.002	-71.4	-17.2
Level of Income	-0.136	0.238	-198.7	49.9
Level of Education	-0.357	0.002	-422.2	-96.6
Total number of barriers	0.085	0.363	-47.5	128.5

## Discussion

To obtain the cardiovascular health benefits, moderate intensity activities should be performed for 30 minutes/day for five or more days of the week. [22] According to the estimates of WHO, the prevalence of physical inactivity among adults is 17% and it ranges from 11% to 24% across different regions in the world. [23] In the present study, 37% of the participants under 60 year age group and 44% of the participants in the age group ≥ to 60 years were not engaging in sufficient levels of moderate intensity physical activities (150 minutes/week). The physical inactivity prevalence of the study is higher than the values stated by the WHO. This is an important and significant public health problem that requires further investigation and action.

### Personal barriers

In the present study, the main personal barriers cited by the respondents were lack of time (52%), laziness (26%) and tiredness (8%) (Table 2). A qualitative study done in India New Delhi involving 14 adult participants in the age range 18–72 years stated that the most common barrier was lack of time (Chandra and Nongkynrih, 2019). [24] According to a study conducted in four cities in Japan involving 865 adults in the age range of 20–69 year, the strongest perceived barrier was lack of time (Ishii et al., 2009). [25] Both these studies done in Asian countries support our study findings with regard to lack of time being the strongest barrier for regular physical activity participation.

A study conducted in the United States on 18–65 year old people has revealed that lack of time, being tired, obtaining enough exercise at the job and no motivation to exercise were the commonest personal barriers. [26] In our study laziness was one of the main barriers for regular physical exercise. Contrary to the findings of our study where laziness was revealed as a common barrier, research conducted in the USA states otherwise. However, no motivation to exercise was cited as a barrier in the study conducted in the United States, and the laziness can be one factor for lack of motivation. In the present study, only 4% of the participants stated that they felt they were getting sufficient physical activity and that there was no need to engage in further exercises. Out of them > 50 were females over the age of ≥ 60 years. In our study, the majority of females over the age of 60 years, stated that they took part in regular household chores, took care of grandchildren and therefore, the physical exercises they were getting was adequate.

According to a study conducted in Australia, among those aged between 60 to 78 years, injury or poor health were the most frequently cited barriers for participation in regular physical activity. [27] In the present study, in the age group ≥ 60 years, poor health was not one of the main barriers for participation in regular physical activity. This could be due to the fact that we excluded people with chronic ill health and painful joint lesions from the study.

Lack of time, feeling too tired and low level of income were barriers for regular physical activity participation among adult females. [28] In conclusion, our study draws parallels with previous studies and justifies many previously identified reasons for lack of participation in physical exercises. In this study, feeling too lazy to get involved in physical exercises was cited by 26% of the participants (Table 2), which is not common in other studies. Therefore, this study reiterates that investing in programmes to motivate people to take part in regular physical activities would be beneficial. In the present study, having a high level of income was associated with low levels of physical activity. This could be due to using motor vehicles to travel to work, rather than walking or cycling. Our study finding with regard to the level of income is different to the findings of Ribeiro and Milanez, (2011) [28].

In our study, subjects with high level of education had a significant negative association with the level of physical activity participation in the age group ≥ 60 years. A cross sectional study done on Nepalese adults in the age range of 15–69 years demonstrates that high level of education, had a negative association with physical activity levels. The reason for high level of education to have a negative association with physical activity were believed to be due to people with high level education in Nepal were involved in more sedentary occupations (Pedisic et al., 2019). [29] This study done in Nepal supports our study findings. As in Nepal, In Sri Lanka too, the people with high level of education may be involved in more sedentary type of occupations.

### Environmental barriers

According to the study done in India by (Chandra and Nongkynrih, 2019), the environmental barriers for regular physical exercise were lack of maintenance of infrastructure and equipment of the gymnasium, lack of cleanliness and uneven ground surface in the parks, lack of outdoor and indoor spaces close to the house and unfavourable weather conditions during different seasons [24]

A qualitative study done on 12 elderly Chinese people states that lack of age appropriate physical activity facilities, available space, peer motivation and general support are the barriers that need to be overcome to improve the physical activity levels of elderly Chinese people (Yanling et al., 2013) [30].

A study done in Dhaka Bangladesh involving adults in the age range of 20–35 years mentions that the most common environmental barriers for regular physical activity participation was poor street lighting at night (62%), and lack of convenient places (56%). The other barriers cited in this study were unclean environments and bad weather (Uddin et al., 2018) [31].

The main environmental barriers reported by studies done in in disadvantaged cities of countries such as the United States, the United Kingdom, New Zealand and Australia were fear of personal safety, lack of access to facilities and bad weather. [8] These environmental barriers have been reported by females and children. These studies do not mention the environmental barriers affecting adult males living in those cities.

In our study, bad weather (29%) (Table 2) was the main environmental barrier cited for physical activity participation by respondents in both age groups. Sri Lanka has a high rain fall and this may be a reason why bad weather was the main environmental barrier identified.

Fear of personal safety due to hazards from thieves, unleashed dogs and snakes (3%) and lack of access to facilities (5%) were cited by only a small percentage of respondents in our study (Table 2). Our study has looked into the environmental barriers that were affecting both males and females in the age groups < 60 years and ≥ 60 years (Table 2). Our study was done in a semi urban area. Therefore, the prevalence of barriers on personal safety and lack of facilities was low. Having cites for physical exercises with adequate safety and protection from rain could help in the motivation of many people to take part in regular physical activities.

### Social barriers

Different cultural beliefs and difficulties in speaking English were the main social barriers for participation in physical activity of South Asian men and women living in developed Western countries. [12] There were no studies done on the South Asian region to find out the cultural barriers for physical activity participation. In our study, the main social barrier cited was that they did not want to be seen by others while being engaged in recreational physical exercise. This barrier was cited only by 7% of females and 2% of males in the age group ≥ 60 years (Table 2). In our study, the majority of participants (91%) belong to the main ethnic (Sinhalese) group and the main language in Sri Lanka is Sinhala. Many people in Sri Lanka are able to speak Sinhala language as it is the most widely used. These could be the reasons why the social barriers such as language difficulties and different cultural beliefs were less cited in our study.

### Factors associated with level of physical activities

Being a male and being younger was positively associated with level of physical activity participation. [32] A study conducted by the Australian Bureau of Statistics (hereafter ABS) found that there was a greater prevalence of physical inactivity among female immigrants from most regions compared to male immigrants from the same or similar regions. It was believed that lack of time due to a women's role in domestic and child raising activities may be contributing for women to be less active than their male counterparts. [33] In our study in ≥ 60 year age group the male gender had a significant negative association (P = 0.016) with physical activity participation and in < 60 year age group gender did not have a significant association with physical activity participation (Table 3). In our study, moderate levels of physical activities in females were greater than the males in both age groups. Our study findings differ from the study findings of Koeneman et al (2011). [32] and Dassanayake et al (2011). [33] In the present study, in both age groups, lack of free time was the main barrier to take part in regular leisure time physical activities among females (Table 1). In <60 year age group, lack of free time was the main barrier to take part in regular leisure time physical activities in males (Table 1) and in ≥ 60 year age group, feeling too lazy was the main barrier to take part in regular leisure time physical activities (Table 2). In this study, 96% of females in < 60 year age group and 80% of females in ≥ 60 year age group were involved in household activities like (cooking, washing the dishes, cleaning the house) and looking after children in addition to the work of their occupation. These physical activities contribute to their daily physical activities and these could be some of the reasons why, in the present study, females had a greater level of physical activities than the males. Therefore, educating and motivating the males who are physically inactive to take part in household work activities will help to increase their physical activity levels.

In our study people with high level of education and high level of income in the age range of 60 years and above had a significant negative association with physical exercise. Contrary to our study findings Shaw and Spokane (2008) states that the level of physical activity decreased with increasing age and this decline was greater among individuals with low education levels. [34] However, according to Abrante set al (2011) the level of education did not have a significant effect on regular physical activity participation. [35] Heide et al (2013) states that people with a low level of education have poor health levels and it is due to poor health literacy levels. They also mention that poor health literacy level is common even among people with high level of education. [36] In the present study people with high level of education did not take part in regular physical activities due to lack of time due to their tight work schedules and people with high level of income used their personal motor vehicles to travel even short distances. According to Matthew et al (2014) psychological stress is associated with lesser levels of physical activities or exercise. [37] In the present study, although the psychological stress levels were not assessed, people with a high level of education involved in occupations that has tight work deadlines may be affected with increased stress levels. These reasons, in addition to lack of time and use of motor vehicles to travel even short distances, may be contributing to low levels of physical activities among people with high level of education and high level of income in the age group equal to above 60 years. People in the age group < 60 years level of education did not have a significant negative association. This could be due to the reason that most of them being involved in house hold work and activities such as gardening.

In this study people with high BMI had a significant negative association with the level of physical activities. According to a study done on overweight females, laziness was the main cause that prevented them from being physically active. [38] In our study 57% of the respondents were overweight and 26% of people cited laziness as a personal barrier for taking part in regular physical activities. Therefore, motivation programmes to reduce laziness and promote regular physical activity participation will help the majority of people to be healthy and maintain their BMI in the normal range.

## Conclusions

This study conducted in Sri Lanka covers a wide area related to physical activities such as levels of physical activity, barriers for physical activity and factors associated with regular physical activity participation. Many studies done in other countries have not compared the barriers and other factors affecting physical activity participation among different age groups. In the present study having a high level of education and being a male was associated with less physical activity levels which are contrary to the findings of previous studies done in other countries. This study reiterates the importance of motivation programmes, educating people on time management skills and having safe environments suitable for physical activity participation. The findings of the present study are significant and useful in designing physical activity promotion programmes for people < than 60 years in age and ≥ 60 years in age for the prevention and treatment of non-communicable diseases of Sri Lankans and for other South Asian people living in their own countries and living in other countries of the world.

This study was done in the Western province, and Sri Lanka has nine provinces. In the Central, Northern and Eastern provinces there are significant differences with regard to the weather, geography and ethnic groups compared to the Western province. Therefore, similar studies need to be carried out in Central, Northern and Eastern provinces since there can be variations in personal, environmental and social barriers for physical activity participation.

## Supporting information

S1 File Data on level of physical activities, education, income, BMI and barriers. (XLSX)
